# Structural Insights into the Mechanism of Protein O-Fucosylation

**DOI:** 10.1371/journal.pone.0025365

**Published:** 2011-09-26

**Authors:** Erandi Lira-Navarrete, Jessika Valero-González, Raquel Villanueva, Marta Martínez-Júlvez, Tomás Tejero, Pedro Merino, Santosh Panjikar, Ramon Hurtado-Guerrero

**Affiliations:** 1 Institute of Biocomputation and Physics of Complex Systems, University of Zaragoza, Zaragoza, Spain; 2 Department of Biochemistry and Molecular and Cellular Biology, University of Zaragoza, Zaragoza, Spain; 3 Department of Organic Chemistry, Institute of Chemical Synthesis and Homogenoeus Catalysis (ISQCH), University of Zaragoza-CSIC, Zaragoza, Spain; 4 EMBL Hamburt Outstation, Hamburg, Germany; 5 Fundación ARAID, Diputación General de Aragón, Zaragoza, Spain; Institute of Molecular and Cell Biology, Singapore

## Abstract

Protein O-fucosylation is an essential post-translational modification, involved in the folding of target proteins and in the role of these target proteins during embryonic development and adult tissue homeostasis, among other things. Two different enzymes are responsible for this modification, Protein O-fucosyltransferase 1 and 2 (POFUT1 and POFUT2, respectively). Both proteins have been characterised biologically and enzymatically but nothing is known at the molecular or structural level. Here we describe the first crystal structure of a catalytically functional POFUT1 in an apo-form and in complex with GDP-fucose and GDP. The enzyme belongs to the GT-B family and is not dependent on manganese for activity. GDP-fucose/GDP is localised in a conserved cavity connected to a large solvent exposed pocket, which we show is the binding site of epidermal growth factor (EGF) repeats in the extracellular domain of the Notch Receptor. Through both mutational and kinetic studies we have identified which residues are involved in binding and catalysis and have determined that the Arg240 residue is a key catalytic residue. We also propose a novel S_N_1-like catalytic mechanism with formation of an intimate ion pair, in which the glycosidic bond is cleaved before the nucleophilic attack; and theoretical calculations at a DFT (B3LYP/6-31+G(d,p) support this mechanism. Thus, the crystal structure together with our mutagenesis studies explain the molecular mechanism of POFUT1 and provide a new starting point for the design of functional inhibitors to this critical enzyme in the future.

## Introduction

Protein O-fucosylation is an important post-translational modification first reported 36 years ago which is mediated by two glycosyltransferases, Protein O-fucosyltransferase 1 (POFUT1) and Protein O-fucosyltransferase 2 (POFUT2) [1], [2]. Both enzymes are inverting glycosyltransferases classified in the CAZy database as GT65 and GT68, respectively [3]. An enzymatic assay for POFUT1 was described in 1998 [4] and the gene was cloned in 2001 [5]. Genetic experiments confirmed POFUT1 is essential during development in both flies and mice [6]–[8] and the phenotypes of the null embryos demonstrated that it is implicated in the Notch signalling pathway. The identity and characterisation of POFUT2 was published later in 2006 and confirmed the existence of these two different glycosyltransferases involved in Protein O-fucosylation [9], [10].

This modification only occurs in eukaryotic organisms and is a rare form of O-linked glycan on cysteine-rich proteins motifs [11]–[13]. Both POFUT1 and POFUT2 act on proteins containing the cysteine-rich motifs as the acceptor sugar and GDP-fucose as the donor [4], [10]. While POFUT1 activity is increased in the presence of metals [4], POFUT2 does not require a metal for catalysis [10]. Unlike the majority of glycosyltransferases, which are localised in the Golgi apparatus, both enzymes reside in the endoplasmic reticulum. However, while POFUT1 contains the typical endoplasmic reticulum localisation sequence, “RDEF” [14], this is not present in the POFUT2 sequence [10].

The most recognised substrates of POFUT1 are the EGF repeats present in the extracellular domain of Notch proteins [5], [11], [14], [15]. Notch is a membrane-anchored signalling receptor which plays very pleiotropic and essential roles during the development of many tissues including regulation of cell fate, proliferation, apoptosis, differentiation and migration [16]. The extracellular domain of Notch harbours 36 EGF repeats in mammals; of those, 23 contain a tetrasaccharide unit in which the first sugar is a fucose [16]–[18], and 20 contain a trisaccharide unit in which the first sugar is a glucose [19]. EGF repeats are small (∼40 amino acids) cysteine-rich motifs with six conserved cysteines forming three disulphide bridges [20], [21]. POFUT1 α-fucosylates serines or threonines present in EGF repeats containing the appropriate consensus sequence, C^2^-X_(4-5)_-[S/T]-C^3^, where C^2^ and C^3^ are the second and third conserved cysteines. Of these fucosylated EGF repeats, EGF11-12 are the most important biologically since this is the region to which the Notch ligands bind [22]–[23]. As mentioned before, fucose is the first residue of a tetrasaccharide found in some EGF repeats followed by an N-Acetyl glucosamine (GlcNAc) residue catalysed by the glycosyltransferase Fringe proteins [24]–[25]. This GlcNAcylation together with Protein O-fucosylation has been shown to be essential in different organisms. The role of GlcNAc is to mediate or increase the interaction of Notch with its ligands [24]–[25]. An additional role of POFUT1 as a chaperone in the folding and secretion of Notch is inferred from disruption studies in flies [8]–[26], unlike in mice, where similar studies do not support this role [27].

Notch is up-regulated in a large number of diseases such as T cell acute lymphatic leukaemia (T-ALL) [28] and adult T-cell leukaemia [29]. Thus the development of pharmacological inhibitors against POFUT1 may provide an alternative strategy to treat these diseases through attenuation of Notch signalling pathway.

Less is known about POFUT2. It recognises a second type of cysteine-rich motif, known as a thrombospondin type 1 repeat (TSR), which contains six conserved cysteines and three disulphide bonds [30]. Literature reports to date would suggest that there are more than 40 proteins affected by this modification, including thrombospondin 1 and 2, the ADAM family of metalloproteases and properdin [31]–[32]. So far the main role of POFUT2 has been suggested to be involved in secretion of different proteins [31]–[32].

Despite considerable interest in the role of these proteins during Notch signalling in embryogenesis, we do not know anything about the crystal structure of these enzymes or how they recognise their substrates or what catalytic mechanism these enzymes employ to modify their target proteins.

In order to address these issues, we describe the first crystal structure of a eukaryotic POFUT1, in native form and in complex with GDP-fucose and GDP. The structures show the typical GT-B folding and a non-metal dependency. Surface electrostatic potential plus docking studies with POFUT1 and human EGF12 (*Hs*EGF12) suggest the EGF repeat binding site. Furthermore, through site directed mutagenesis experiments we propose the catalytic and binding residues involved in Protein O-fucosylation. Interestingly we also suggest the role of a conserved Arg240 as a key catalytic residue, which facilitates the glycosidic cleavage that occurs prior to proton transfer of the acceptor substrate to the catalytic base, β-phosphate. This work provides a molecular framework for further studies towards POFUT1 specificity and for the design of novel POFUT1 inhibitors.

## Results and Discussion

### *Caenorhabditis elegans* POFUT1 (*Ce*POFUT1) is a catalytically functional enzyme which is not dependent on manganese for activity

**Figure 1:**
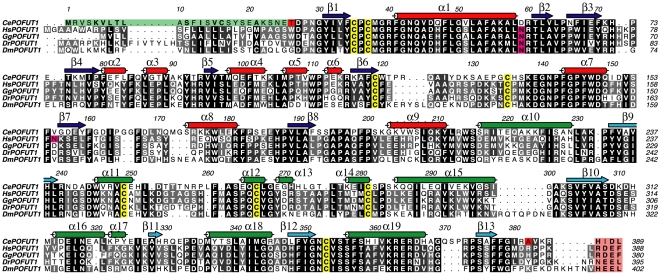
Multiple sequence alignment of POFUT1s. Multiple sequence alignment of the GT65 family members *Ce*POFUT1, *Hs*POFUT1, *Gallus gallus* POFUT1 (*Gg*POFUT1), *Danio renio* POFUT1 (*Dr*POFUT1) and *Dm*POFUT1. Secondary structure elements from the *Ce*POFUT1 structure are shown, with α-helices in red and green for the N and C-terminal domains, respectively, and β-strands correspondingly in blue and cyan. Signal sequence of *Ce*POFUT1 is highlighted in green while endoplasmic reticulum retention sequence for all POFUT1s are indicated in a pink box. Conserved cysteines forming disulphide bridges and N-glycosylated sites are shown in yellow and magenta, respectively. The boundaries of the N and C-terminal *Ce*POFUT1 constructs are indicated in red for Thr26 and Ala382.

**Figure 2:**
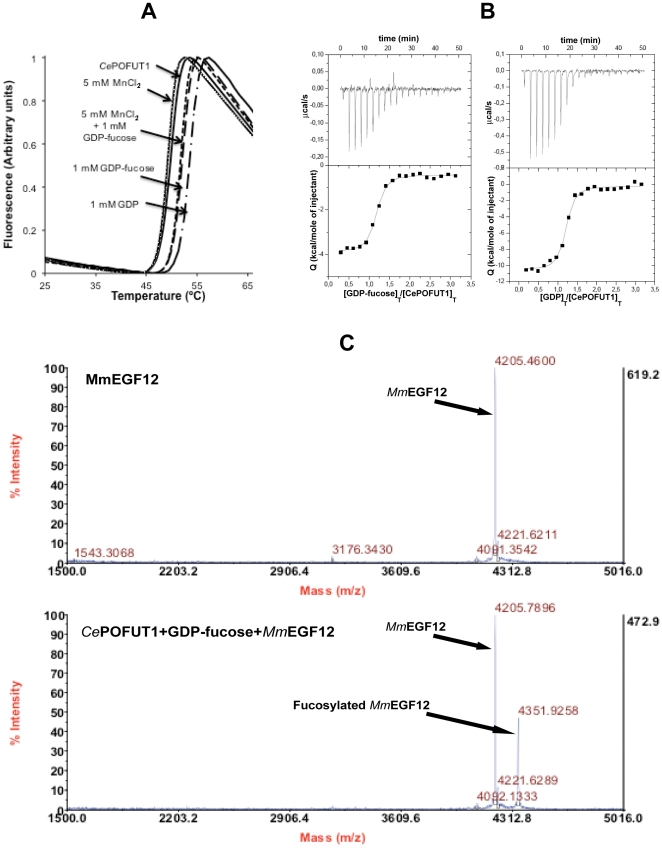
Biophysical characterisation of *Ce*POFUT1. (**A**). Thermal denaturation curves of the wild type enzyme as monitored by ANS fluorescence in the absence or presence of 5 mM MnCl_2_, 1 mM GDP-fucose/GDP and 1 mM GDP-fucose together with 5 mM MnCl_2_. (**B**). ITC experiments of wild type enzyme on GDP-fucose and GDP. (**C**). MALDI-TOF MS of *Mm*EGF12 as a control and incubated with wild type enzyme and GDP-fucose. The mass of *Mm*EGF12 and fucosylated *Mm*EGF12 are indicated by arrows.

*Ce*POFUT1 was identified and selected by a protein blast search from different organisms. Unlike its homologues in other species it lacks N-glycosylation sites [33] (**Figure 1**). This protein showed ∼41% identity with higher eukaryote enzymes (**Figure 1**), suggesting it functions as a protein O-fucosyltransferase 1. Based on a multiple alignment (**Figure 1**), we expressed a truncated form of *Ce*POFUT1 (amino acids 26–382, excluding the signal sequence and the retention endoplasmic reticulum localisation sequence, **Figure 1**) as a secreted protein in *Pichia pastoris* and we purified it by a HiTrap-Blue, ion exchange and by gel filtration chromatography (see **Materials and Methods**). POFUT1s have been shown to bind GDP-fucose and EGF repeats, and transfer this monosaccharide into small EGF repeats producing GDP during the reaction. Moreover manganese has been shown to increase the transfer activity of these glycosyltransferases on EGF repeats [4], [5]. In order to test the binding of *Ce*POFUT1 to GDP-fucose/GDP and manganese, we conducted a thermal shift-assay [34] and isothermal titration calorimetry experiments (**Figure 2A and 2B**). The protein showed a denaturation temperature *T*_0_ of 50.07±0.09°C, which was increased to 2.2 and 4.2°C by incubation of the enzyme with 1 mM GDP-fucose and GDP, respectively (**Figure 2A** **and** **Table 1**), suggesting binding to the donor sugar and GDP. Analysis by isothermal calorimetry titrated by different concentrations of the ligands (**Figure 2B**) confirmed quantitatively the thermal denaturation data rendering a *K_d_* (dissociation constant) of 0.23±0.04 µM for GDP-fucose (which is in the low µM range and similar to the *K*_m_s of 4 and 6.4 µM reported for *Drosophila melanogaster* and mouse POFUT1 [4], [5], respectively) and 0.35±0.07 µM for GDP (**Table 2**). Since both GDP-fucose and GDP bind with a similar affinity to the enzyme, inhibition by-products may occur as has been described for other types of glycosyltransferases [35], [36]. As expected for a highly negatively charged ligand, the binding energy is dominated, for example in the case of GDP, by a large negative entalphic term (ΔH =  −10.37±0.22 kcal/mol) compared to a non-favoured entropic term (T*ΔS  =  −1.6 kcal/mol). Furthermore, in both GDP-fucose and GDP, the stochiometry observed experimentally was in agreement with a single binding site. Unlike nucleotides, experiments with 5 mM MnCl_2_ both with and without GDP-fucose showed small decreases of 0.25–0.45°C in the Δ*T*_m_ compared to the wild type enzyme (**Figure 2A**), indicating that manganese may slightly decrease the stability of *Ce*POFUT1.

**Table 1:** *T*_0_ and *ΔT*_m_ of mutants compared with wild type enzyme.

	*T*_0_ (°C)	*ΔT*_m_ (°C)1 mM GDP	*ΔT*_m_ (°C)5 mM GDP	*ΔT*_m_ (°C)1 mM GDP-fucose
Wild Type	50.07±0.09	4.21	6.17	2.19
R40A	50.94±0.11	0.85	2.71	-
N43A	51.07±0.17	2.04	-	1.20
F199A	48.04±0.13	2.94	-	-
R240A	52.77±0.10	0.1	0.09	-
R240K	50.92±0.05	1.04	1.27	-
D242A	48.04±0.14	6.81	-	-
D244A	48.52±0.08	4.58	-	-
W245A	49.02±0.15	0.80	2.19	-
F261A	48.77±0.13	4.50	-	1.84
D309N	45.82±0.15	5.01	-	-
F357A	49.65±0.16	0.15	0.90	-

The *T*_0_s represent means±S.D. for three independent experiments.

**Table 2:** GDP-fucose/GDP dissociation constants of mutants compared with wild type enzyme.

	*K*_d_ (µM)GDP	*K*_d_ (µM)GDP-fucose
Wild Type	0.35±0.07	0.23±0.04
R40A	3.10±0.53	-
N43A	0.30±0.09	-
F199A	0.18±0.03	-
R240A	nd	-
R240K	61.2±10.5	-
D242A	0.09±0.02	-
D244A	0.10±0.01	-
W245A	1.60±0.40	-
F261A	0.12±0.07	-
D309N	-	-
F357A	26.9±5	-

The *K*_d_s were determined from thermodynamic relationships and the estimated errors were less than±24% in the majority of the cases. n.d.  =  not detected; -  =  not determined.

To evaluate the enzymatic properties of *Ce*POFUT1, we characterised its activity as a glycosylhydrolase and glycosyltransferase. The hydrolase activity was measured by an enzymatic coupled assay with NDPase, rendering a specific activity value of 90±8 pmol/min*mg in the presence of 100 µM GDP-fucose. We also determined the activity of *Ce*POFUT1 in the presence of 1 and 5 mM MnCl_2_, showing that the enzyme is inhibited by manganese (between 40–60% inhibition, data not shown), contrary to what has previously been described [4]. The transferase activity assay was carried out with the enzyme, GDP-fucose and *Mus musculus* EGF12 (*Mm*EGF12) from the mouse Notch1 receptor protein, and determined by MALDI mass spectrometry (**Figure 2C**). Strikingly an expected increase of 146 Daltons was obtained in the fucosylated peptide, in accordance to a fucose bound to *Mm*EGF12 (**Figure 2C**). Therefore, *Ce*POFUT1 is a Protein O-fucosyltransferase 1 which acts independantly of manganese and which recognises the EGF12 repeat from mouse Notch1.

### *Ce*POFUT1 adopts a GT-B fold

**Figure 3:**
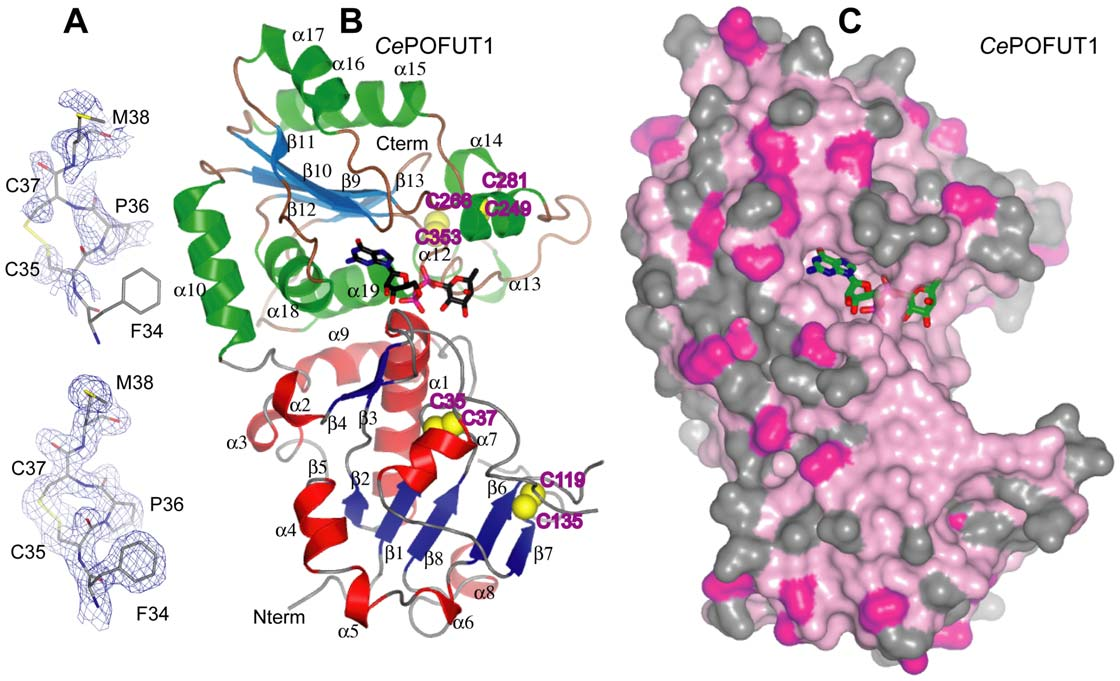
Overall crystal structure of *Ce*POFUT1. (**A**). 2|F_o_ |-|F_c_ | electron density maps for the region around the first disulphide bridge, Cys35-Cys37. 2|F_o_ |- |F_c_ |, f_calc_ electron density maps are shown at 1.0 σ. The upper map was obtained from initial phases from SHELXC/D/E [52] while the map below corresponded to improved phases obtained from phase extension (see **Materials and Methods**). (**B**). Overall crystal structure of *Ce*POFUT1 in complex with GDP-fucose. Secondary structure elements from the *Ce*POFUT1 structure are shown, with α-helices in red and green for the N and C-terminal domains, respectively, and β-strands correspondingly in blue and cyan. The four disulphide bridges are highlighted in yellow. GDP-fucose is shown as sticks with black carbon atoms for illustration purposes. (**C**). Surface representation of the *Ce*POFUT1, colored by sequence conservation ((pink (100% identity), to grey (<50% identity)). GDP-fucose is shown as sticks with green carbon atoms.

*Ce*POFUT1 is classified in the CAZy database [3] as GT65. Currently there is no available data for the structure or folding properties of this protein. To determine the structure of *Ce*POFUT1 we obtained three different crystal forms. Due to the reproducibility and diffracting properties of crystals-form-II (see **Materials and Methods**), we solved the structure from a highly redundant crystal-form-II, previously soaked with MnCl_2_, by sulphur SAD experiments, in combination with phase extension from a crystal co-crystallised with GDP and soaked with GDP-fucose/MnCl_2_ diffracting at high resolution (**Figure 3A** **and** **Table 3** for data collection and refinement statistics). Iterative model building and refinement yielded an initial model which was used to solve crystal-form-I and III with good refinement statistics (R = 0.234, R*_free_* = 0.252 for native crystal-form-I and R = 0.204, R*_free_* = 0.244 for crystal-form-III in complex with GDP, **Table 3**). Soaking experiments of crystal-form-I and II with GDP-fucose yielded a complex to 1.96 Å (R = 0.218, R*_free_* = 0.267, **Table 3**) and 1.91 Å (R = 0.202, R*_free_* = 0.237, **Table 3**).

**Table 3:** Data collection and refinement statistics.

	*Ce*POFUT1 (apo-form),crystal-form-I	*Ce*POFUT1 and GDP-fucose,crystal-form-I	*Ce*POFUT1 and GDP (High redundant dataset),crystal-form-II	*Ce*POFUT1, GDP and Mn^+2^ (High resolution dataset),crystal-form-II	*Ce*POFUT1 and GDP-fucosecrystal-form-II	*Ce*POFUT1 and GDP,crystal-form-III
Space group	C2	C2	C2	C2	C2	P6_5_
Number of molecules in the Asymmetric unit (AU)	1	1	1	1	1	2
Wavelength (Å)	0.97	1.54	1.54	0.99	1.54	0.99
Resolution (Å)	20.0–1.74(1.83-1.74)	56.01–1.96(2.06-1.96)	36.66-2.60(2.70-2.60)	19.50–1.54(1.62-1.54)	20.00–1.91(2.01-1.91)	24.98–1.86(1.96-1.86)
Cell dimensions (Å)	*A* = 0117.44*b* = 042.53 *c* = 96.92	*a* = 0117.54*b* = 042.53 *c* = 97.66	*a* = 0144.26*b* = 037.97*c* = 67.95	*a* = 0145.01*b* = 038.25 *c* = 68.20	*a* = 0144.58*b* = 038.15 *c* = 68.10	*a* = 0132.18*b* = 132.18 *c* = 77.56
Unique reflections	46747	42027	21952	53812	39386	64057
Completeness	98.9 (96.6)	98 (94.7)	100 (100)	99.1 (99.7)	98.9 (96.5)	98.8 (97.4)
*R*_sym_	0.114 (0.280)	0.113 (0.484)	0.106 (0289)	0.036 (0.281)	0.096 (0.517)	0.067 (0.618)
*I*/σ(*I*)	6.5 (2.7)	12.19 (2.60)	52.23 (17.87)	20.8 (4.8)	9.27 (1.97)	22.7 (3.1)
Redundancy	2.8 (2.7)	7.06 (5.01)	77.87 (59.88)	4.1 (4.0)	4.37 (3.91)	9.4 (6.7)
*R*_work_/*R*_free_	0.234/0.252	0.218/0.267		0.205/0.237	0.202/0.237	0.204/0.244
RMSD from ideal geometry, bonds (Å)	0.011	0.013		0.011	0.012	0.011
RMSD from ideal geometry, angles (°)	1.361	1.432		1.373	1.375	1.364
<*B*> protein (Å^2^)	27.03	23.08		22.85	26.61	27.47
<*B*> GDP-fucose (Å^2^)		22.28			25.38	
<*B*> GDP (Å^2^)				17.00		18.76
<*B*> solvent (Å^2^)	28.25	23.16		31.23	29.18	29.56
Ramachandran plot: Most favoured (%)Additionally allowed (%)Generously allowed (%)	97.941.470.59	97.441.710.85		97.622.080.30	96.822.600.58	97.661.750.58
PDB ID	3ZY4	3ZY5		3ZY2	3ZY6	3ZY3

Values in parentheses refer to the highest resolution shell. Ramachandran plot statistics were determined with PROCHECK [55].

*Ce*POFUT1 is a monomeric protein, as determined by gel filtration chromatography and confirmed by analytical ultracentrifugation (data not shown). This protein is composed of two domains (**Figure 3A**) which is a conserved feature shared by all higher eukaryotic POFUT1s (**Figure 1** **and** **3A**). The N and the C-terminal domains (α1- α9/β1- β8 and α10- α19/β9- β13, respectively) adopt Rossmann-like folds, which are formed by a central β-sheet surrounded by α-helices on both sides and these constitute the typical signature of a GT-B fold. The donor sugar, GDP-fucose, is localised in the interface where the two domains face each other; a typical signature of glycosyltransferases adopting the GT-B fold (**Figure 3A**). The residues from the active site interacting with GDP-fucose are mainly from the C-terminal domain (**Figure 3A**) together with a few from the N-terminal domain. Glycosyltransferases adopting GT-B folds are metal-independent [37] and this characteristic is further supported by the thermal denaturation curves and kinetic experiments shown above (**Figure 2** **and** **3**). A structure search on the DALI [38] server revealed not surprisingly structural homology with two other fucosyltransferases, nodulation fucosyltransferase [39] (NodZ) (RMSD [Root mean square deviation] on 231 equivalent Cαs =  4.1 Å) and α-1,6 fucosyltransferase or FUT8 [40] (RMSD on 222 equivalent Cαs =  3.7 Å), which also contain the typical GT-B fold. The structure with the third highest Z score was ADP-heptose LPS heptosyltransferase II (RMSD on 184 equivalent Cαs =  3.8 Å) (PDB ID 1PSW), which folds as a GT-B and is functionally different to fucosyltransferases.

A comparison among the different crystal forms revealed minor conformational changes within the two domains (RMSD of 0.25 Å among apo crystal-form-I and soaked with GDP-fucose, and 0.66–0.86 Å among crystal-form-I compared to crystal-form-II and III, respectively) and some differences in order/disorder loop changes, such as a loop in the N-terminal domain (residues 125–134). The larger RMSD could have resulted from differences in crystal packing and not from bound ligands due to the small RMSD among crystals-form-I with and without the ligand.

*Ce*POFUT1 contains four conserved disulphide bridges through the GT65 family (**Figure 1** **and** **3A**), two in each domain. The first one, placed in a loop connecting β1 and α1 and formed by Cys35 and Cys37 (**Figure 3A**), is an unusual disulphide bridge due to the proximity between both cysteines and represents a signature of the all family (**Figure 1**). This loop contributes to form the sugar donor-binding site together with residues from α1 and it is possible that this disulphide bridge helps to correctly position them. The second one is formed between Cys139 from β6 and Cys135 placed in a loop preceding α7. This disulphide occurs in the vicinity of disordered loops and it is possible that it helps to limit flexibility. Cys249 and Cys281 form the third disulphide, which comes from the α11 and α14, respectively. α11 and the previous loop contribute to the sugar donor binding site with key amino acids (**Figure 1** **and** **3A**). The fourth disulphide, formed by Cys266 and Cys353, comes from the α12 and the loop between β12 and α19, respectively. Amino acids in the loop preceding α12 contribute to the sugar donor-binding site (**Figure 1** **and** **3A**).

### *Ce*POFUT1 is a good representative of the GT65 family

**Figure 4:**
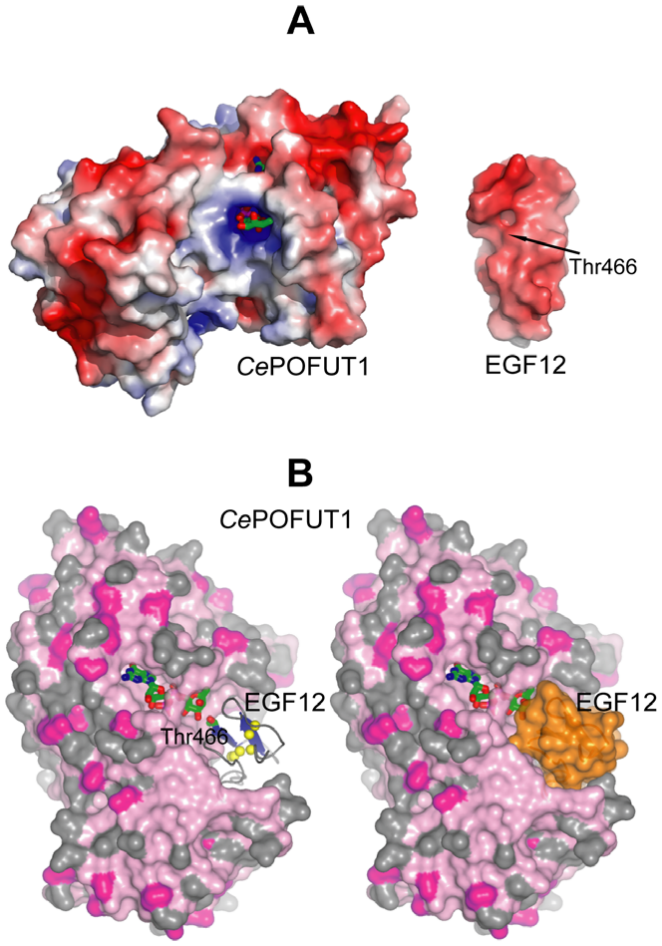
Localisation of EGF repeat binding site in *Ce*POFUT1. (**A**). Electrostatic surface representation of *Ce*POFUT1 and *Hs*EGF12 (PDB code 2VJ3). *Ce*POFUT1 has an overall positive charge in the sugar and acceptor binding site while *Hs*EGF12 is negatively charged around Thr466 [56]. GDP-fucose is shown as sticks with green carbon atoms while Thr466 is indicated by an arrow. (**B**). Docking of *Hs*EFG12 into *Ce*POFUT1. *Ce*POFUT1 is represented as a surface model colored by sequence conservation, while *Hs*EGF12 is shown either as ribbon representation on the left (β-strands are in blue and the three disulphide bridges are highlighted in yellow) or as a surface model (in orange) on the right. GDP-fucose and Thr466 are shown as sticks with green carbon atoms.

The crystal structure and the high identity between *Ce*POFUT1 and the sequence of POFUT1 in higher eukaryotics (identity ≥41%), implies structural conservation of GT65 family members (**Figure 1** **and** **3B**) and suggests that the sugar donor binding site may be completely conserved (**Figure 1** **and** **3B**). Furthermore GDP-fucose points out to a very conserved solvent exposed pocket, in which EGF repeats are expected to bind (**Fig. 3B** **and** **4**). To confirm the binding site of EGF repeats, we analysed the electrostatic surface potential (**Figure 4A**) between the *Hs*EGF12 repeat from human Notch and *Ce*POFUT1. The results showed that sugar nucleotide donor and the acceptor binding site were overall positive while EGF12 was negatively charged (**Figure 4A**), suggesting electrostatic complementarity between both proteins. Furthermore, docking studies with ClusPro [41] were carried out to confirm binding site and orientation of the EGF repeats (see **Materials and Methods, and** **Figure 4B**). EGF repeats contain two antiparallel β-strands and the glycosylated Ser/Thr residues preceding the third cysteine are localised at the beginning of the first β-strand. We analysed the 30 top structures returned by ClusPro (the *Hs*EGF12 was located in the pocket mentioned above in all of the output structures), and we selected two complexes (**Figure 4B**) based on the Thr466 orientation with respect to the sugar nucleotide GDP-fucose. Surprisingly, in only one of these two complexes, Thr466 OG1 is 5 Å from the fucose anomeric carbon, suggesting not only the right localisation but also the right orientation of *Hs*EGF12. Furthermore, Thr466 is facing the sugar from the α-face, which is compatible with the inverting character of POFUT1.

Therefore *Ce*POFUT1 provides a useful tool with which to understand the catalytic mechanism of GT65 family members and to provide a platform to develop future inhibitors against human POFUT1 in order to treat diseases in which Notch signalling activity is up-regulated [28], [29], due to its high structural identity with the human enzyme.

### GDP-fucose is bound in a conserved cavity formed mainly by amino acids from the C-terminal domain

**Figure 5:**
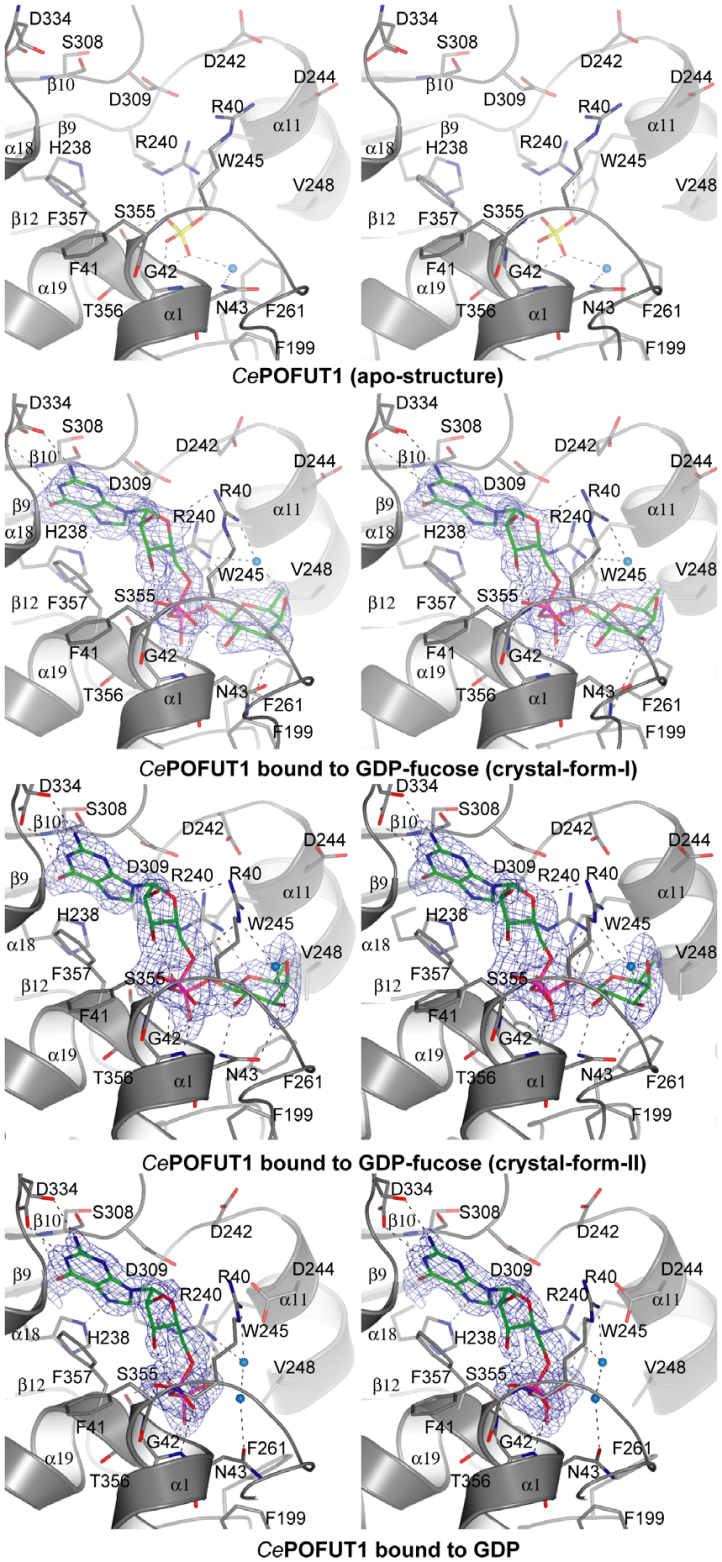
Active site of *Ce*POFUT1 apo-form and in complexes with GDP-fucose/GDP. Stereo view of the active site of *Ce*POFUT1 apo-form, in complex with GDP-fucose and GDP. The amino acids placed in the active site are shown as sticks with grey carbons. GDP-fucose and GDP are represented as stick models with green carbon atoms. Protein-ligand and water-ligand hydrogen bonds are shown as dotted black lines. Only water molecules localised in the fucose binding site are shown for clarity purposes. Water molecules are shown as cyan spheres. β and α faces of fucose are indicated to understand the stereochemistry of the catalytic reaction. Unbiased (i.e. before inclusion of any ligand model) |F_o_ |- |F_c_ |, f_calc_ electron density maps are shown at 2.2 and 2.5 σ.

The four crystal structures described in the current manuscript represent three different steps during catalysis (**Figure 5** **and Figure S1**). The first two structures were obtained by crystals-form-I. One of them represents the apo-form bound to a sulfate molecule in the active site, while the second structure is bound to GDP-fucose resembling the substrate binding mode. The third one is a crystal-form-II, in complex with GDP-fucose (also resembling the substrate binding mode), which contains amino acids in the active site with different conformational changes in relation to the above structure. The fourth one is a crystal-form-III in complex with GDP, resembling a product complex. In the structures complexed with ligands, GDP-fucose and GDP occupied identical positions (**Figure 5**). The nucleotide was localised between α1, α18, α19, β10, β12 and loops among β1- α1, α15- β10, β11- α18 and β12- α19 (**Figure 5**). While guanosine and fucose adopt favourable conformations, the pyrophosphate group of GDP-fucose/GDP shows torsion angles (α, β, γ are 61°, 48°, and −2°, respectively) distorted from ideal angles, which are 180^o^ (**Figure 5**). Unusual torsion angles are also found in *Helicobacter pylori* α-1,3-fucosyltransferase (*Hp*FucT) in complex with GDP-fucose [42] (α, β, γ are −60°, 138° and −78°, respectively), suggesting that fucosyltransferases twist pyrophosphate in order to fit GDP-fucose into their active sites. Due to these changes, two intra hydrogen bonds are formed in GDP-fucose/GDP, one between the ribose O3 and the α-phosphate opposite oxygen, and the second one between fucose O2 and the α-phosphate opposite oxygen (**Figure 5**). The guanine ring is sandwiched between Phe357 and Asp309 by stacking hydrophobic interactions (in the case of Asp309, α and β carbons of this residue contribute to the hydrophobic interaction) (**Figure 5**). The rest of the interactions with guanine occur through hydrogen bonds with backbones of Ser308 and Asp234, and side chains of His238 and Asp334 (**Figure 5**). The ribose ring makes hydrogen bonds with the Arg40 side chain and the Phe41 backbone (**Figure 5**). The α-phosphate interacts through hydrogen bonds with the Gly42 and Asn43 backbones while β-phosphate makes hydrogen bonds with the Ser355, Thr356 and Arg240 side chains and also shows an additional salt bridge with the latter amino acid (**Figure 5**). In the apo structure, a sulphate group occupies the β-phosphate position found in the complex with GDP-fucose and maintains conserved interactions with Arg240 and Thr356. The fucose is recognised by a stacking hydrophobic interaction with Phe261, and hydrogen bonds through its oxygen ring with Arg240, and O3, O4 with the Asn43 side chain (Asn43 makes different hydrogen bonds with fucose depending on its conformations in the crystal structures, **Figure 5**). Moreover, there is an additional interaction between fucose O4 and a water molecule in the β-face (only water molecules around the fucose binding site will be discussed, just for clarification purposes), which also interacts with Arg40. Finally, a second water molecule from the apo structure is present in the fucose α-face, making hydrogen bonds with sulphate and Asn43. This water molecule may represent the catalytic incoming water or Ser/Thr of the EGF repeat (to be discussed later, **Figure 5** **and** **6**). Several conformations are seen for Arg40, Asn43, Arg240, Asp242, Asp244 and Phe261 in the above mentioned structures suggesting flexibility in the catalytic region.

### Site-directed mutagenesis studies suggest Arg240 as a key residue involved in binding and catalysis

On the basis of these crystal structures, we evaluated the importance of several amino acids in the binding and catalytic properties of this enzyme by site-directed mutagenesis. We mutated residues involved in binding to guanosine (Phe357, Arg40, Asp309), pyrophosphate (Arg240), fucose (Asn43, Arg240 and Phe261) and also around the ligand, but not directly implicated in binding such as Phe199, Trp245, Asp242 and Asp244. Phe199 was close to Asn43 and Phe261, while Trp245 was hydrophobically stacked to Arg240. Inverting glycosyltransferases requires an aspartic, glutamic acid or histidine as a catalytic base to deprotonate an incoming acceptor substrate in order to attack the anomeric carbon bound to the nucleotide [43]. Consequently, we mutated the only two acidic amino acids, Asp242 and 244, which were close to the sugar donor binding site.

We firstly characterised them by thermal denaturation curves, showing two different groups of amino acids, both less and more stable than the wild type. R40A, N43A and R240A/K were more stable while F199A, D309N, D242A, D244A, W245A, F261A and F357A were less stable (**Table 1**). We further incubated them with increasing concentrations of GDP to compare the Δ*T*_m_ with that of the wild type in the same conditions (**Table 1**). F199A, D242A, D309N, D244A, F261A showed increases similar to the wild type at 1 mM GDP; N43A was half of the wild type Δ*T*_m_; while R40A, R240A/K, W245A and F357A had less than 4-fold reduction in the Δ*T*_m_. Δ*T*_m_ of the latter mutants was increased with 5 mM GDP except for R240A, which did not show any significant change. Finally both mutants, N43A and F261A, had a decrease in Δ*T*_m_ at 1 mM GDP-fucose compared to that of the wild type (**Table 1**). These results suggest that mutated amino acids with less increase in the Δ*T*_m_ compared to the wild type, may show decreased binding to the nucleotide. In order to confirm this hypothesis, we further evaluated the binding of these mutants by ITC experiments. All the mutants were titrated with GDP. Three different groups of mutants were found in relation to the binding of GDP. N43A and F199A bound to GDP with similar *K_d_* (**Table 2**) to the wild type, in accordance with a non direct interaction of these amino acids with the nucleotide (**Figure 5**). A second group of mutants such as R40A, R240A/K, W245A and F357A showed a decrease in binding to GDP (**Table 2**). From this group, R40A and W245A bound better to GDP than F357A, R240K and R240A, with the latter being impaired in binding under our conditions (see **Table 2** **and** **Materials and Methods**). These results suggest that Arg40 and Trp245 may have an important structural role in the active site, while Phe357 and Arg240 are critical amino acids in binding to the nucleotide (**Figure 5**). A third group, which includes mutants such as D242A, D244A and F261A, showed 3-fold improvement in binding, although none of these residues interact directly with GDP.

**Figure 6:**
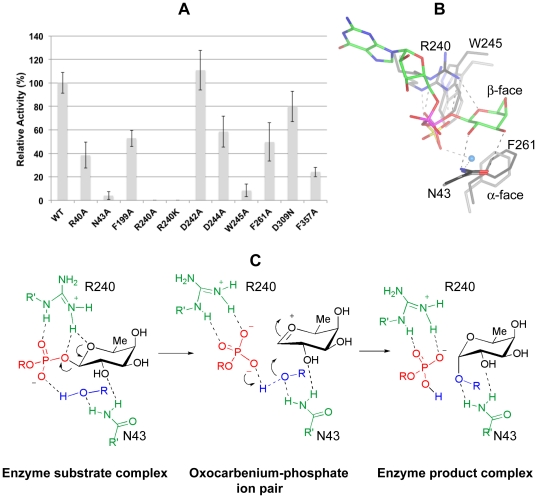
Site-directed mutagenesis, superposition study and proposed catalytic mechanism. (**A**). Glycosylhydrolase activity assay of mutants compared with that of the wild type enyme. The activity of mutants is shown as relative to the wild type enzyme (being 100%). The data represent means±S.D. for six to nine independent experiments. (**B**). Superposition of a GDP-fucose complex into the apo-form structure. Asn43, Arg240, Trp245 and Phe261 are shown as sticks with grey carbons. GDP-fucose is represented as stick models with green carbon atoms. A water molecule localised in the α-face is shown as a cyan sphere. Dotted hydrogen bonds between Asn43 and Arg240 from the apo structure and sulphate are shown as grey while the same amino acids from the complex and GDP-fucose are shown as black. Asn43, Arg240, Trp245 and Phe261 from both structures superpose with a RMSD of 0.21 Å, 0.77 Å, 0.91 Å and 0.29 Å, respectively. Sulphate of apo structure mimics β-phosphate of GDP-fucose because adopts an analogous position. (**C**). S_N_1-like catalytic mechanism with formation of an intimate ion pair in the transition state. R in blue is the hydrogen of a water molecule or the rest of the incoming EGF repeat. Arg240 and Asn43 are in green, GDP is in red, fucose is in black and the incoming acceptor substrate is in blue. The proton transferred during the reaction is in blue. Protein-ligand and incoming acceptor substrate-ligand hydrogen bonds are shown as dotted black lines.

On the other hand we evaluated the hydrolase activity of these mutants on GDP-fucose (**Figure 6A**). D309N and D242A showed a very similar activity to the wild type enzyme while D242A, R40A, F199A, D244A, F261A and F357A displayed a slight decrease in activity. Finally the activity of W245A and N43A was diminished 12.5 and 25-fold, respectively, while R240A/K were impaired catalytically (**Figure 6A**). These results suggest that the majority of the mutated amino acids may affect activity due to changes in their ability to bind the nucleotide or due to their influence in the nature of the folding of the active site. Only Asn43, Arg240 and Trp245 were critical for activity.

### Catalytic mechanism

To our surprise, the activity data with D242A and D244A suggested that the enzyme may not have any amino acid acting as a catalytic base. Unlike Asp242 or Asp244, Arg240 played an important key role in binding and catalysis. Under the conditions assayed, R240A was completely impaired with respect to binding to GDP while R240K was 175-fold decreased with respect to binding to GDP. R240K may have better binding than R240A since R240K may mimic the positive charge present in Arg240. Strikingly, although R240K showed a reduced binding to GDP, the activity was completely abolished. Our data with Arg240 are supported by mutagenesis studies of an equivalent Arg245 in *Drosophila melanogaster* POFUT1 (*Dm*POFUT1, **Figure 1**) which is also present in α-1,2 and α-1,6 fucosyltransferases. In these enzymes, mutations of this key arginine also impaired the activity [44]-[46]. Therefore Arg240 (Arg245 in *Dm*POFUT1) plays a conserved and critical function in POFUT1 which is also extended to other fucosyltransferases.

A detailed analysis of the active sites of the crystal structures (**Figure 5**), together with a superposition of the GDP-fucose complex on the apo structure (**Figure 6B**), suggests that Arg240, through a hydrogen bond with the glycosidic bound oxygen, may facilitate the cleavage of the glycosidic bond. The stacking hydrophobic interaction between Arg240 and Trp245 may explain the decreased affinity and activity of W245A. Finally, Asn43 is a flexible amino acid involved in binding to fucose (**Figure 5**) and it is also important in catalysis. The superposed structures show that Asn43 may be critical in positioning the incoming water or Ser-Thr residue of the EGF repeat in order for catalysis to take place. Certainly this suggestion would fit with our data showing its importance in catalytic activity. Based on the superposition study we also propose β-phosphate as the catalytic base due to a possible hydrogen bond with the incoming water/Ser-Thr of the EGF repeat (see hydrogen bond between sulphate and water molecule in the apo structure, **Figure 5**). Thus, the model would be that the cleavage of the glycosidic bond would take place first; and an oxocarbenium ion would be formed leading to a transition state with an ion pair. In this scenario, Arg240 should maintain the salt bridge and hydrogen bond to β-phosphate but not to the fucose ring oxygen, in order for the oxocarbenium ion to be stable (Arg240 would make the oxocarbenium ion unstable if the interaction was maintaned). Furthermore, the β-phosphate group would associate closely with the incipient oxocarbenium ion blocking the upper face and allowing the backside attack of the incoming acceptor substrate. This mechanism is also favoured by the concomitant proton transfer from the acceptor substrate to the phosphate group as illustrated in **Figure 6C**. The cleavage of the glycosidic bond before the attack to the anomeric carbon has been also suggested for an inverting α-1,3 fucosyltransferase V [47].

In order to support this proposal, we explored the mechanism of the reaction by using DFT methods at a B3LYP/6-31+G(d,p) level (see **Materials and Methods**, **and** **Figure S2 and S3**). the transition structure TS-1 was located after an exploration of the potential energy surface guessed as a function of the predefined reaction coordinates based on X-ray data. The analysis of the geometrical change occurring along the reaction pathway demonstrates that the phosphate-anomeric carbon bond is broken leading to the formation of an oxacarbenium ion pair with the concomitant entry of the water molecule and without the formation of any intermediate. In the transition structure both geometrical parameters and frequency analysis confirm the above proposed mechanism. Analyzing the located transition state it is possible to see the breaking of the bond between the anomeric carbon and the β-phosphate. The length of such a bond is 1.40 Å in the reactants increasing to 3.18 Å at the TS; however the oxygen phosphate involved in proton transfer remains at 2.63 Å indicating a stabilizing electrostatic interaction. The attack of the water molecule is facilitated by the hydrogen bond with the phosphate group leading to a distance between the water oxygen and the anomeric center of 2.72 Å at the TS. In this transition state the proton transfer occurs between the water molecule and the phosphate group. The role of Arg240 is crucial in order to stabilize the charge of the incipient free phosphate group thus promoting its dissociation from the anomeric center.

### Concluding Remarks

Despite the biological importance of Protein O-fucosylation as a post translational modification event in both embryogenesis and adult homeostasis, there has not been a single study devoted to the molecular or catalytic mechanism of the enzymes involved in this modification event. Furthermore, although POFUT1 and POFUT2 fucosylate a large number of acceptor substrates with different functions, both enzymes still recognise similar types of small domains or repeats. Thus, POFUT1 and POFUT2 may follow a similar folding and catalytic mechanism. In the present study we have solved the first crystal structure of *Ce*POFUT1, which serves as a conserved family member.

*Ce*POFUT1 is a catalytically competent non manganese-dependent enzyme, transfering fucose to *Mm*EGF12. The protein shows a typical GT-B fold formed by two Rossmann domains. A cavity for GDP-fucose is localised in the interface between the domains and a contiguous solvent exposed pocket for EGF repeats was identified by electrostatic and docking studies. Mutagenesis studies on several amino acids suggest the importance of Arg240, Phe261 and Phe357 in binding to the sugar nucleotide, and Asn43 together with Arg240 as important catalytic residues. The absence of an amino acid as a catalytic base and the presence of a water molecule interacting with a sulphate in the fucose α-face of the apo structure, suggest that β-phosphate may represent the catalytic base. Thus, we propose that Arg240 facilitates the cleavage of the glycosidic bond with formation of a oxocarbenium ion, prior to a proton transfer from the incoming acceptor substrate to the leaving phosphate and consequently attacking of the acceptor substrate to the anomeric carbon. In this mechanism Asn43 may position the hydroxyl group of the incoming substrate close to the β-phosphate. We further support this mechanism by means of theoretical calculations at a DFT level, which clearly indicated the formation of an ion pair at the transition state, between the incipient oxocarbenium ion and the phosphate group, in which the charge transfer is facilitated by the residue Arg240. The reaction can be considered to follow a S_N_1-like mechanism even though the proton transfer between the water and the phosphate group takes place with the entry of the nucleophile and the formation of the ion pair, and without formation of any intermediate.

Furthermore these studies may represent a significant advance for the design and development of future compounds which may be useful to treat diseases in which Notch signalling activity is up-regulated.

## Materials and Methods

### Cloning, expression and purification

The DNA sequence encoding amino acid residues 26–382 of the *Caenorhabditis elegans Ce*POFUT1 (swissprot: locus OFUT1_ CAEEL; accession Q18014; GeneID: C15C7.7), defined as *cepofut1*, was made synthetically and codon optimized by GenScript for expression in *Pichia pastoris*. The DNA, containing at the *5*′ end a recognition sequence for *Xho*I and a KEX2 cleavage signal and at the *3*′ end a sequence for *Sac*II, was cloned into the pUC57 vector (GenScript). Following digestion with *Xho*I and *Sac*II the cloned sequence was subcloned into the *Pichia pastoris* protein expression and secretion vector pPICZαA (Invitrogen), resulting in the expression plasmid pPICZαA*cepofut1* (T26-A382).

The plasmid pPICZαA*cepofut1* (T26-A382), referred to here as the wild type, was used as a template for introducing the following single amino acid changes by site-directed mutagenesis: R40A, N43A, F199A, R240K, R240A, D242A, D244A, W245A, F261A, D309N, F357A, such that each of the resulting 11 plasmids carried the indicated mutation. Site-directed mutagenesis was carried out following the ‘QuikChange’ kit protocol (Stratagene), using the KOD HotStart DNA polymerase (Novagene). All plasmids were verified by sequencing (Sistemas Genómicos, Servicio de Secuenciación; www.sistemasgenomicos.com).

All plasmids were isolated from the *E. coli* strain DH5α, linearised with *Sac*I and used to transform the *Pichia pastoris* strain into X-33 by electroporation. Transformants were selected on YPD plates (1% (w/v) yeast extract, 2% (w/v) peptone, 2% (w/v) dextrose) containing 100 µg/ml of zeocin (InvivoGen). Batch cultures were performed in 100 ml volume of BMGY medium (1% (w/v) yeast extract, 2% (w/v) peptone, 100 mM potassium phosphate (pH 6.0), 1.34% (w/v) yeast nitrogen base and 1% (v/v) glycerol). 50 ml were used to grow 500 ml of BMGY medium overnight at 30°C and expression was induced by methanol (1%, v/v) for 72–96 h at room temperature in a shaking incubator (270 rpm). Yeast cells were harvested by centrifugation at 3480 g for 30 min. The supernatants containing soluble *Ce*POFUT1 were filtered to 0.45 and 0.2 µm, concentrated to 20–50 ml using a Pellicon XL device (10,000 MWCO, PES membrane; Millipore) and dialyzed against 25 mM Tris pH 8.5.

The samples were then loaded onto a 1×5 ml HiTrap Blue Sepharose (Amersham Biosciences) that had been equilibrated with 10 column volumes of 25 mM Tris pH 8.5 on an AKTA purifier system. Following loading, the column was washed with 10 column volumes of 25 mM Tris pH 8.5. The protein was eluted with a salt gradient (0–1 M NaCl) over 15 column volumes, collecting 3 ml fractions. The fractions containing the proteins were then pooled and dialyzed against 25 mM Tris pH 8.5.

The dialysed sample was loaded onto a 1×5 ml HiTrap Q FF column (Amersham Biosciences) that had been equilibrated with 10 column volumes of 25 mM Tris pH 8.5 on an AKTA purifier system. Following loading, the column was washed with 10 column volumes of 25 mM Tris pH 8.5. The protein was eluted with a salt gradient (0–1 M NaCl) over 15 column volumes, collecting 3 ml fractions. The fractions containing the proteins were then pooled and concentrated to 2.5 ml using centrifugal filter units of 10,000 MWCO (Millipore). Subsequently, gel filtration was carried out using a Superdex 75 XK26/60 column in 25 mM Tris, 150 mM NaCl, pH 8.5. The concentrated *Ce*POFUT1 protein, previously dialysed in 25 mM Tris pH 8.5, was used for both kinetic analysis and crystallization trials.

### Thermal shift ligand binding assays

To monitor the binding of ligands to *Ce*POFUT1, the thermal-shift assay was performed. The method is based on the observation that ligands change protein thermal stability upon binding to the protein. This results in a change of the midpoint temperature for the thermal protein unfolding transition, *T*_m_.

The protein unfolding process is monitored using the environmentally sensitive dye 1-anilino-8-naphthalene sulfonate (ANS). Its quantum yield increases upon binding to hydrophobic surfaces exposed during protein unfolding and so does the fluorescence signal.

The thermal-shift assay was conducted in the FluoDia T70 (Photal Otsuka Electronics). Solutions of 2 µM *Ce*POFUT1, 100 µM ANS, 20 mM HEPES pH 7.0, and 200 mM NaCl final concentration and a volume of 100 µl were added to the wells of a 96-well PCR plate. Mineral oil was last added to avoid evaporation during the experiment. The final concentration of the ligands were: GDP-fucose/GDP at 1 and 5 mM, MnCl_2_ at 5 mM, and GDP-fucose with MnCl_2_ at 5 mM. Mutants were also evaluated under the same conditions.

Data were analysed with the Origin software. To obtain *T*_m_, a Boltzmann model from Origin was used to fit the fluorescence imaging data:

I  =  (A+B ((B-A)/(1+exp (Tm-T)/C))

where *I* is the fluorescence intensity at temperature *T*, *A* and *B* are pretransitional and posttransitional fluorescence intensities, respectively, and *C* is a slope factor.

Data points after the fluorescence intensity maximum were excluded from fitting. In the absence of ligands, *T*_m_ = *T*_0,_ and the ligand-dependent changes in midpoint temperature, Δ*T*_m_ = *T*_m_−*T*_0_, could be calculated for each ligand.

### Isothermal titration microcalorimetry (ITC)

ITC was used to evaluate the dissociation constants of *Ce*POFUT1 and mutants against GDP-fucose and GDP. All experiments were carried out at 25°C with concentrations of *Ce*POFUT1 and mutants between 10 and 30 µM, and concentrations of GDP-fucose and GDP between 150 µM and 1 mM, in 25 mM Tris, pH 7.5.

The reactions were performed on an AUTO ITC instrument Microcal Auto-iTC_200_. Data integration, correction and analysis were carried out using Origin 7 (Microcal) with a single-site binding model.

### Hydrolysis of GDP-fucose by *Ce*POFUT1

*Ce*POFUT1 activity was assayed by testing its ability to hydrolyse GDP-fucose. 5 µM *Ce*POFUT1 was incubated with 100 µM GDP-fucose and 0.01 µg/ml NTPDase 3 (R&D Systems) in 25 mM Tris, 5 mM CaCl_2_, pH 7.5 to a final volume of 36 µl. Mixtures were incubated for 3 and 20 hours at 25°C. The reaction was stopped by heating the samples at 80°C for 10 min. Release of inorganic phosphate was measured with the Malachite Green Phosphate Detection kit (R&D Systems) and the absorbance was read at 620 nm with a microplate reader from Biotek sinergy HT. 100 µM GDP-fucose and 100 µM GDP with 0.01 µg/ml NTPDase 3 were also assayed as controls. The same protocol was used to assay *Ce*POFUT1 mutants' activity.

### Mass spectrometry analyses

A folded synthetical *Mm*EGF12 repeat was purchased from JPT Peptide Technologies GmbH, in order to determine the transfer activity of *Ce*POFUT1. The *Mm*EGF12 repeat, at <70% purity, was incubated with 5 µM *Ce*POFUT1 and 100 µM GDP-fucose in 25 mM Tris, 5 mM CaCl_2_, pH 7.5, for 20 hours at 25°C. A sample of *Mm*EGF12 repeat in the same buffer was taken as a control.

The samples were concentrated and desalted by passing them through ZipTip C18 columns (Millipore) following the manufacturer's instructions and eluting with 70% acetonitrile (ACN), 0.1% trifluoroacetic acid (TFA) in water.

Sample (0,4 µl) and matrix (0,8 µl saturated solution of alpha-*Cyano*-4-hydroxycinnamic acid in 50% ACN, 0.1% TFA in water) were spotted in duplicate onto a Opti-Tof 384 well insert plate (Applied Biosystems). MALDI-TOF MS was performed using a 4800plus MALDI-TOFTOF (Applied Biosystems) in the reflector negative mode with accelerating voltage of 20 kV, mass range of 1000 to 5000 Da, 500 shots/spectrum and laser intensity of 2800. Spectra were calibrated externally using bovine insulin (+2) m/z 2867,8, bovine insulin (+1) m/z 5734,6.

### Crystallization and data collection

*Ce*POFUT1 was spin-concentrated to 30 mg/ml. Three different crystal forms corresponding to two different space groups (**Table 3**) were grown by sitting drop experiments at 18°C through mixing 1 µl of protein with an equal volume of a reservoir solution. Native/apo crystals, belonging to C2 space group (crystal-form-I), were obtained in 100 mM BIS-TRIS, 2 M ammonium sulphate, pH 6.0. The other C2 crystals (crystal-form-II) obtained by cocrystallisation among the protein and 5 mM GDP were grown from 100 mM HEPES, 100 mM MgCl_2_, 20% PEG3350, pH 7.5. To obtain the second space group, P6_5_ (crystal-form-III), the protein was cocrystallised as described above, and crystals were produced from a solution containing 100 mM HEPES, 2% PEG 400, 1.8 M ammonium sulphate, pH 6.5. The crystals grown in ammonium sulphate were cryoprotected with saturated lithium sulphate, while the ones grown in PEG3350 were cryoprotected in the same mother liquor plus 35% PEG3350, and flash cooled prior to data collection at 100 K. GDP-fucose complex was generated by soakings of native and crystal-form-IIs with 10–100 mM GDP-fucose in mother liquor for 10–20 minutes prior to data collection (crystal-form-II at high resolution was also soaked with 100 mM MnCl_2_). The latter crystal soaked with this high concentration of metal contains a manganese atom localised in the fucose position and appears to be coordinated by β-phosphate group oxygen atom of GDP together with two water molecules (**Figure S1**). We think that this is probably an artefact due to the high amount of the metal used and thus does not have any significant function.

Highly redundant sulphur SAD and a native crystal soaked with GDP-fucose data were collected in house on a Bruker microsource with a Kappa goniometer and an Axiom detector, all other data were collected at beamline BM16 and ID23-1 (ESRF, Grenoble). All data were processed and scaled using the XDS package [48], the PROTEUM suite and CCP4 software [49], relevant statistics are given in **Table 3**.

### Structure determination and refinement

By sulphur single anomalous diffraction (SAD) phasing methodology, using highly redundant data from a crystal cocrystallised with GDP (**Table 3**) and using the software pipeline Auto-Rickshaw [50], [51], SHELXC/D/E [52] identified 17 sites, yielding initial phases to resolution 2.6 Å. Iterative phase improvement was conducted with an isomorphous high resolution data set of crystal-form-II at 1.54 Å to generate initial maps (to be described elsewhere), from which a model for the apo structure was built with ARP/warp [53] (initially building 315 out of 346 residues of the single protein monomer in the asymmetric unit) and improved through cycles of manual model building in Coot [54] and refinement with REFMAC5 [49]. Molecular replacement with this structure as a search model was used to generate phases and starting models for the remaining data sets, which were refined as described above. Topologies for the oligosaccharide ligands were generated with SKETCHER [49] (part of the CCP4 software). The final models were validated with PROCHECK [55], model statistics are given in **Table 3**. Coordinates and structure factors have been deposited in the Worldwide Protein Data Bank (wwPDB) under accesion codes 3ZY2, 3ZY3, 3ZY4, 3ZY5 and 3ZY6.

### Rigid Body Docking

The *Ce*POFUT1 crystal structure was used for rigid body docking together with *Hs*EGF12 structure obtained form the pdb 2VJ3 [56]. The fully automated online docking program ClusPro was used to dock the structure [41]. The PDB file for the *Ce*POFUT1 crystal structure was submitted as the receptor structure, whereas the PDB file of an *Hs*EGF12 was submitted as the ligand structure. The selected docking program was PIPER, which uses a clustering radius of 9 Å and provides 1000 low energy structures [41], [57]–[59]. The top 30 structures were returned and only one of them was selected based on the orientation with GDP-fucose bound to *Ce*POFUT1. Images were generated using PyMOL.

### Theoretical Calculations

All calculations were conducted using the DFT method, using the B3LYP functional as implemented in Gaussian 09 [60]. The basis set used is the standard all-electron split-valence basis set 6–31+G(d,p). Frequency calculations were conducted to characterize transition state, reagents and products, as well as to use as a basis for determining free energy values at 298 K. Geometry optimizations and vibrational analyses were performed without any coinstraint. On the basis of the existing structural data and to reduce computational cost, a model maintaining the fundamental characters of the real reaction system has been chosen. We used the simplest model with one phosphate unit, which is involved in proton transfer and nucleophilic attack and the presence of the fundamental residue Arg240, modelled as a guanidine unit involved in hydrogen bonding with the phosphate unit. The calculated reaction coordinates are illustrated in **Figure S2** and the located transition structure TS-1 in **Figure S3**.

As discussed in the manuscript, the calculated reaction pathway and the located transition structure confirm the hypothesis outlined from X-ray data. The crucial Arg240 promotes the initial bond breaking between the phosphate group and the anomeric center by forming an incipient oxacarbenium ion, which is stabilized in the form of a ion-pair formed through pivotal of the phosphate unit to approximate the second oxygen atom involved in proton transfer with the nucleophile. Such a situation is favored by the presence of Arg240 which stabilizes the negative charge formed at the oxygen atom previously linked to the anomeric carbon, in addition to a hydrogen bond with the hydroxyl group at C-2 of the fucose unit. Simultaneously, Arg240 goes far from the endocyclic fucose oxygen atom to allow the charge displacement responsible of the formation of an electrophilic anomeric center and, at the same time, the nucleophile approximates such anomeric carbon bringing out the proton transfer to the phosphate group. This situation is captured in the located transition structure TS-1 thus supporting the initial hypothesis concerning the formation of an intimate ion pair in the transition state as well as the only need of the intrinsic phosphate group to promote the reaction.

## Supporting Information

Figure S1 **Stereo view of the active site of the high resolution dataset of** ***Ce*** **POFUT1 in complex with GDP.** The amino acids placed in the active site are shown as sticks with grey carbons. The density suggests the presence of GDP, GDP-fucose and a manganese atom. Due to a partial density for fucose (see black arrows), we decided to include two molecules of GDP. GDP are represented as stick models with green carbon atoms. Manganese is shown as brown sphere and appears to be coordinated by β-phosphate group oxygen atom and two water molecules. Protein-ligand and water-ligand hydrogen bonds are shown as dotted black lines. Only water molecules localised in the fucose binding site are shown for clarity purposes. Water molecules are shown as cyan spheres. Unbiased (i.e. before inclusion of any ligand model) |F_o_ |- |F_c_ |, f_calc_ electron density map is shown at 2.5 σ.
(TIF)

Figure S2 **Calculated Reaction Coordinate.**
(TIF)

Figure S3 Optimized transition structure at a B3LYP/6-31+G** level.
(TIF)
