# Supplementary figures and images for: Structural Insights into the Mechanism of Protein O-Fucosylation
Source: PLoS One. 2011 Sep 26;6(9):e25365. doi: 10.1371/journal.pone.0025365 (PMC3180450; doi:10.1371/journal.pone.0025365)

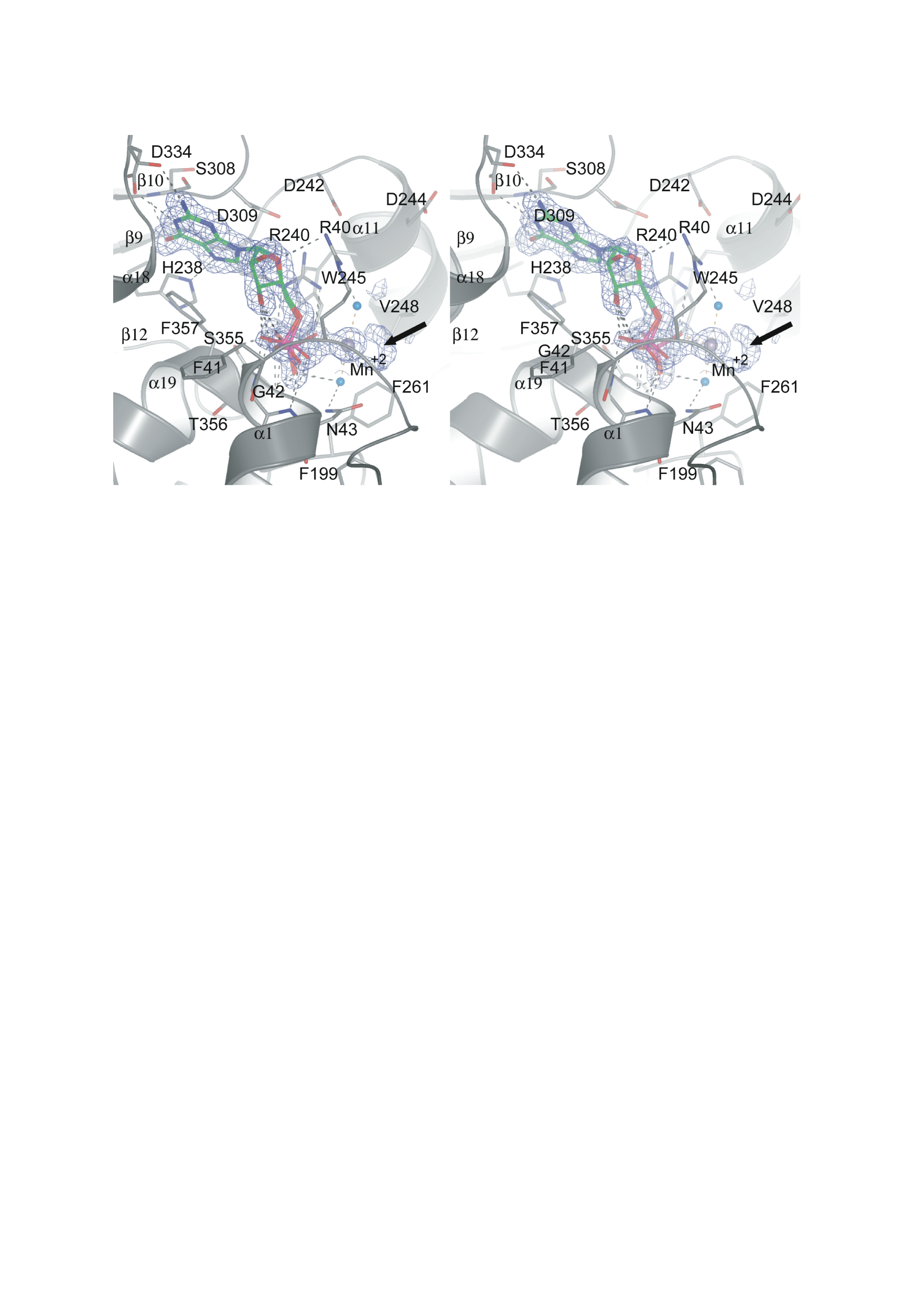

Supplement: Figure S1 — Stereo view of the active site of the high resolution dataset of Ce POFUT1 in complex with GDP. The amino acids placed in the active site are shown as sticks with grey carbons. The density suggests the presence of GDP, GDP-fucose and a manganese atom. Due to a partial density for fucose (see black arrows), we decided to include two molecules of GDP. GDP are represented as stick models with green carbon atoms. Manganese is shown as brown sphere and appears to be coordinated by β-phosphate group oxygen atom and two water molecules. Protein-ligand and water-ligand hydrogen bonds are shown as dotted black lines. Only water molecules localised in the fucose binding site are shown for clarity purposes. Water molecules are shown as cyan spheres. Unbiased (i.e. before inclusion of any ligand model) |Fo |- |Fc |, fcalc electron density map is shown at 2.5 σ. (TIF) [file pone.0025365.s001.tif]

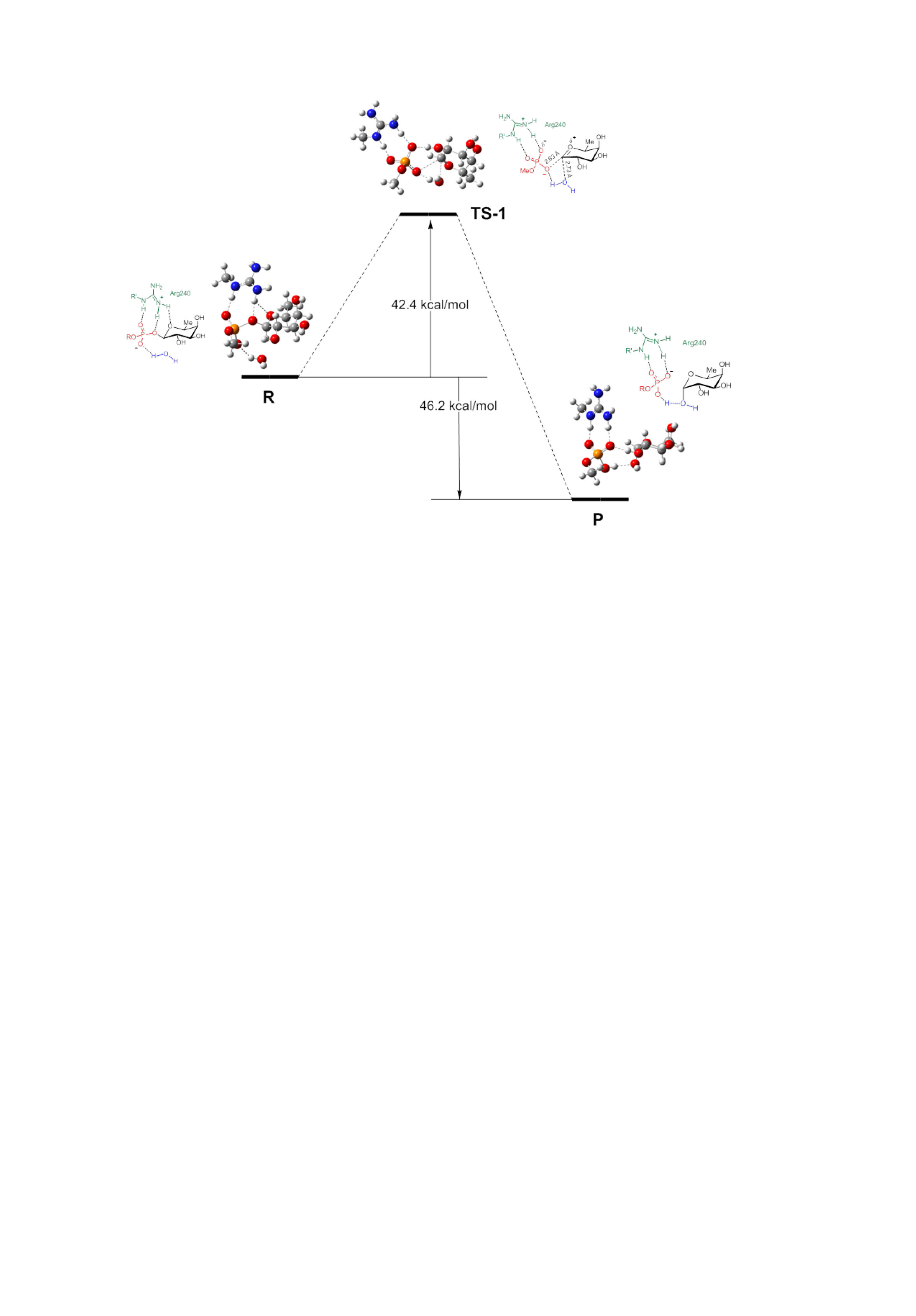

Supplement: Figure S2 — Calculated Reaction Coordinate. (TIF) [file pone.0025365.s002.tif]

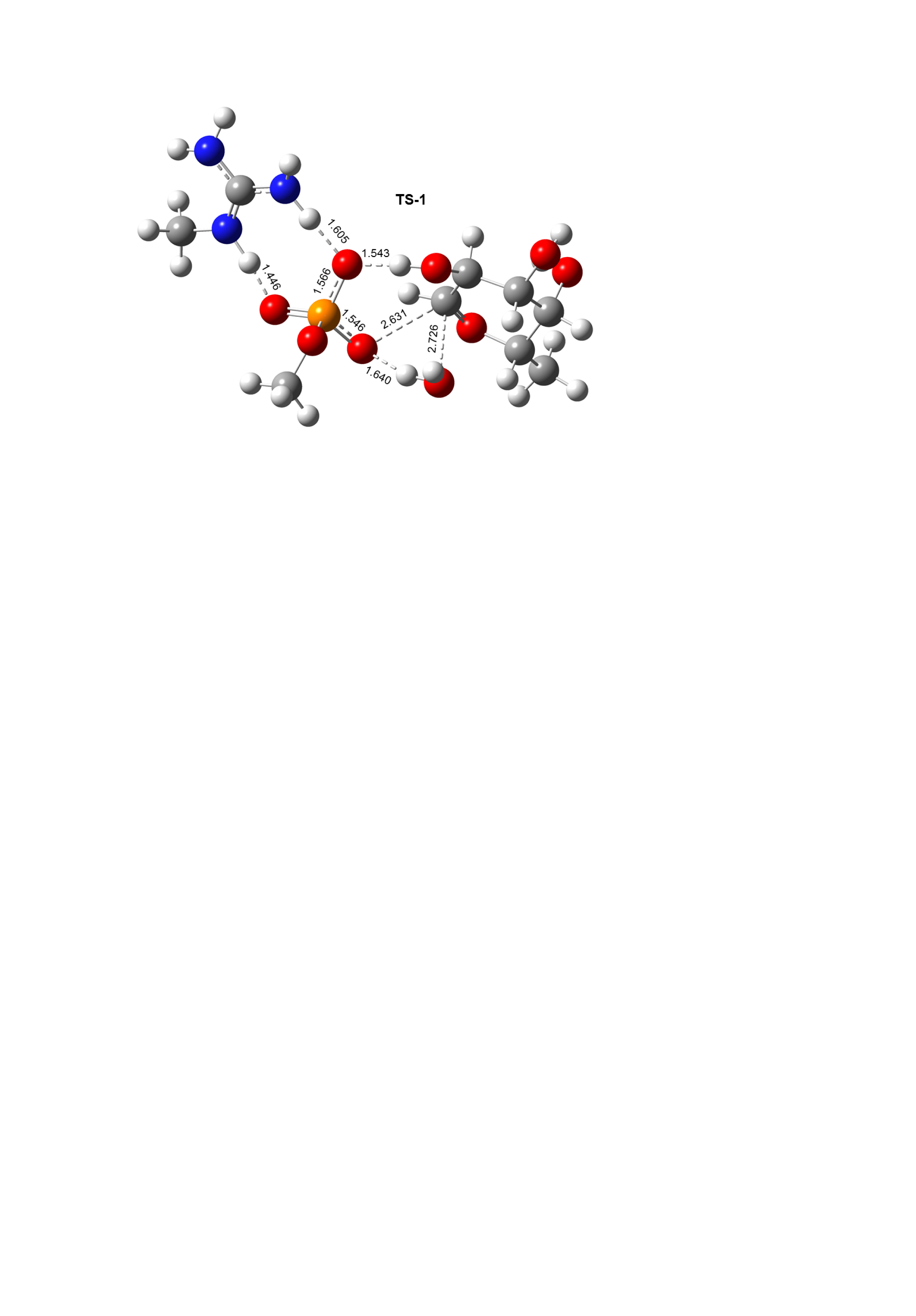

Supplement: Figure S3 — Optimized transition structure at a B3LYP/6-31+G** level. (TIF) [file pone.0025365.s003.tif]
